# Identification of key modules and hub genes for small-cell lung carcinoma and large-cell neuroendocrine lung carcinoma by weighted gene co-expression network analysis of clinical tissue-proteomes

**DOI:** 10.1371/journal.pone.0217105

**Published:** 2019-06-05

**Authors:** Haruhiko Nakamura, Kiyonaga Fujii, Vipul Gupta, Hiroko Hata, Hirotaka Koizumu, Masahiro Hoshikawa, Saeko Naruki, Yuka Miyata, Ikuya Takahashi, Tomoyuki Miyazawa, Hiroki Sakai, Kouhei Tsumoto, Masayuki Takagi, Hisashi Saji, Toshihide Nishimura

**Affiliations:** 1 Department of Chest Surgery, St. Marianna University School of Medicine, Kanagawa, Japan; 2 Department of Translational Medicine Informatics, St. Marianna University School of Medicine, Kanagawa, Japan; 3 The Systems Biology Institute, Tokyo, Japan; 4 Medical Proteomics Laboratory, The Institute of Medical Science, The University of Tokyo, Tokyo, Japan; 5 Department of Pathology, St. Marianna University Hospital, Kanagawa, Japan; 6 Corporate Technology Research and Development, NISSHA Co., Kyoto, Japan; University of Michigan, UNITED STATES

## Abstract

Small-cell lung carcinoma (SCLC) and large-cell neuroendocrine lung carcinoma (LCNEC) are high-grade lung neuroendocrine tumors (NET). However, comparative protein expression within SCLC and LCNEC remains unclear. Here, protein expression profiles were obtained via mass spectrometry-based proteomic analysis. Weighted gene co-expression network analysis (WGCNA) identified co-expressed modules and hub genes. Of 34 identified modules, six were significant and selected for protein–protein interaction (PPI) network analysis and pathway enrichment. Within the six modules, the activation of cellular processes and complexes, such as alternative mRNA splicing, translation initiation, nucleosome remodeling and deacetylase (NuRD) complex, SWItch/Sucrose Non-Fermentable (SWI/SNF) superfamily-type complex, chromatin remodeling pathway, and mRNA metabolic processes, were significant to SCLC. Modules enriched in processes, including signal recognition particle (SRP)-dependent co-translational protein targeting to membrane, nuclear-transcribed mRNA catabolic process of nonsense-mediated decay (NMD), and cellular macromolecule catabolic process, were characteristically activated in LCNEC. Novel high-degree hub genes were identified for each module. Master and upstream regulators were predicted via causal network analysis. This study provides an understanding of the molecular differences in tumorigenesis and malignancy between SCLC and LCNEC and may help identify potential therapeutic targets.

## Introduction

Small-cell lung carcinoma (SCLC) and large-cell neuroendocrine carcinoma (LCNEC) are classified into high-grade lung neuroendocrine tumors (NETs) [1]. In contrast to SCLC, which accounts for approximately 15% of all lung cancers, LCNEC accounts for only approximately 3% of all lung cancers. However, the proportion of pathologically confirmed LCNECs is increasing [2]. The 2009 World Health Organization (WHO) classification of lung tumors classified LCNEC into a specific type of non-small-cell lung carcinoma (NSCLC); however, the revised 2015 WHO classification of lung tumors now classifies LCNEC as a NET [1]. Although some features, specific to their neuroendocrine nature, are common to both SCLCs and LCNECs, their histopathological characteristics are different. SCLC cells typically have a round to fusiform morphology and grow in sheets and nests that frequently include necrotic areas. In addition, these tumor cells have scant cytoplasm, fine chromatin granules, and are less than three times the diameter of small, resting lymphocytes [3, 4]. However, combined SCLCs, including some NSCLC components, can seldom also be found [1]. Similarly, LCNECs show a typical neuroendocrine morphology, including organoid nesting, cellular palisading, or rosette-like structures as well as high mitotic rates. However, they can also manifest cytological features of non-small-cell carcinomas, such as large cells with abundant cytoplasm. From the clinical point of view, these two histological types present similar patient characteristics, including a greater incidence in men (particularly in those who are heavy smokers), diagnosis at an older age, and worse prognosis. Because patients with these histological types are usually discovered only in the advanced stages of the disease, surgically treated patients are rare. A study including 113 patients with SCLC and 141 patients with LCNEC treated with surgical resection reported 5-year all-stage survival rates of 35.7% for SCLC and 40.3% for LCNEC; both showed lower prognosis compared with patients with NSCLC [5]. Because only few studies have evaluated the effectiveness of chemotherapy to date, a standard chemotherapy regimen has not yet been established for LCNEC [6].

Genetic analyses of SCLCs are characterized by mutations of the *RB1* and *p53* tumor suppressors. Loss-of-function mutations are speculated to accelerate the cell cycle, resulting in rapid and continued tumor growth. In contrast, LCNECs are characterized by mutually exclusive *RB1* and *p53* inactivation; however, combinations of frequent mutations in *STK11*/*KRAS*/*KEAP1*, which also occur in NSCLC, are observed [7]. These results indicate that LCNECs may exhibit both neuroendocrine and NSCLC-like features. However, information regarding protein expression in these two types of NETs is limited. Thus, we conducted proteomic analyses to understand the biological characteristics of NETs, including tumor development and differentiation, and to clarify the similarities and differences between SCLCs and LCNECs.

Advancements in high-accuracy mass spectrometry (MS) have rendered proteomics more compatible with shotgun sequencing and quantitative analysis of disease-related proteins obtained from clinical specimens. The resulting data from such analyses are expected to assist in the discovery of novel biomarkers and therapeutic targets [8, 9]. Using the technique of laser microdissection (LMD), it is possible to collect target cells from sections cut out from formalin-fixed paraffin-embedded (FFPE) cancer tissues. Label-free spectral counting and identification-based semi-quantitative shotgun proteomic analysis of microdissected targeted cancerous cells were used in this study [10–14].

The methodology of weighted gene co-expression network analysis (WGCNA) [15] has been effective for the detection of co-expressed modules and hub genes as well as micro- and link-RNAs (long non-coding RNAs) [16–25]. Using this method, expressed genes can be grouped into a model or a network module based on pairwise correlations between genes due to their similar expression profile, and these models can be correlated with the different stages or subtypes of various cancers. A recent comparative study between the WGCNA method and the traditional step-wise multi-marker Cox regression analysis for the simultaneous analysis of multiple tumor expression array markers reported improved validation success of the WGCNA groups and markers [26]. Here, we aimed at identifying clinically significant co-expressed modules and hub proteins/genes, which play key roles in each lung cancer subtype, and used WGCNA for FFPE tissue- proteome datasets of SCLC and LCNEC. This study, to the best of our knowledge, is the first report concerning the application of WGCNA to clinical tissue proteome datasets.

## Materials and methods

### FFPE tissue specimens and sample preparation

Out of the total 974 patients who underwent surgical lung cancer resection at St. Marianna University Hospital between 2000 and 2014, only 41 tumors (4.2%) were histologically confirmed NETs. Pathological specimens were reviewed independently by two pathologists (H.N. and M.T.) to confirm that they satisfied the 2015 WHO classification of lung tumor histological criteria [27]. FFPE tumor tissue blocks from 15 surgical specimens of neuroendocrine tumors, comprising six SCLCs and six LCNECs, were obtained without patient identifiers from St. Marianna University School of Medicine Hospital with informed consent of all participating subjects and under strict Institutional Review Board standards and Ethical Committee approval (Acceptance no. 1461). For tissue microdissection, 10 μm thick sections from the FFPE tumor blocks were cut onto DIRECTOR slides (OncoPlex Diagnostics Inc., Rockville, MD, USA). The sections were de-paraffinized and stained with hematoxylin using standard histological methods prior to dissection. Microdissection was performed using a Leica LMD7 Microdissection Microscope (Leica, Wetzlar, Germany). For combined SCLC specimens, only those tumor cells that showing typical SCLC features were microdissected and analyzed. A total area of 4 mm^2^, consisting of about 15,000 tumor cells, was transferred from the FFPE sections via laser dissection directly into the cap of a 200 *μ*L low-binding tube. Proteins were extracted and digested with trypsin using Liquid Tissue MS Protein Prep kits (OncoPlex Diagnostics Inc., Rockville, MD, USA) according to the manufacturer’s protocol [28]. Details of procedures were described in detail elsewhere [29]

### Liquid chromatography-tandem mass spectrometry based proteomic analysis

A label-free quantitation approach using spectral counting by LC-MS/MS was adopted for a global proteomic analysis. The digested samples (5 *μ*L for a single run) were analyzed in triplicate by LC-MS/MS using reverse-phase LC interfaced with a Q Exactive Orbitrap mass spectrometer (Thermo Fisher Scientific, Bremen, Germany) *via* a nano-ESI device (AMR Inc., Tokyo, Japan). LC-MS/MS analysis was described in detail previously [29].

The raw data were processed using PatternLab for Proteomics software v4.0 [30]. Peptide sequence matching was performed using the Comet algorithm [31] against the UniProt *Homo sapiens* database, downloaded in January 2017. A target-reverse strategy was employed for increased confidence in protein identification [32]. This search considered tryptic peptide candidates, and the formylation of lysine and oxidation of methionine were considered as variable modifications. The Comet search engine considered a precursor mass tolerance of 40 ppm and a fragment bin tolerance of 0.02. The validity of the peptide spectrum matches was assessed using PatternLab’s Search Engine Processor (SEPro) module [33]. Acceptable FDR for spectra, peptide and protein are 3%, 2% and 1%, respectively [29, 34]. The expression level of identified proteins was attained by spectral count-based protein quantification. The spectral count (*SpC*) was the number of MS/MS spectra assigned to each protein. Proteins identified in SCLC and LCNEC were subjected to GO analysis using PANTHER Ver. 11.0 (http://www.pantherdb.org/) [35].

### Construction of gene co-expression networks and identification of modules

Weighted-gene co-expression network analysis (WGCNA) [15] was used to identify systems level differences in protein expression pattern of the lung neuroendocrine subtypes. Using the WGCNA R package [15], pairwise Pearson correlation for all proteins in the dataset was computed and an adjacency matrix was calculated by raising the correlation matrix up to power of 10 (soft thresholding parameter) to generate a scale-free network. By implying a soft thresholding parameter, the weighted gene expression network emphasizes on significantly (high) correlated protein pairs and filters non-significant (low) correlations and, thus reduces the noise of correlation in the adjacency matrix until the network resembles to a scale-free graph. Next, to measure the connection strength between all protein pairs, topological overlap measure (TOM) was calculated from the adjacency matrix. TOM dissimilarity matrix (1-TOM) was then used to perform average linkage hierarchal clustering generating protein clustering tree with modules corresponding to the branches of the tree. Using dynamic tree-cutting the branches were trimmed at 0.99 height so that each module has a minimum number of 10 proteins.

Modules were summarized by the first principal component referred as eigengene in the text. Module membership, defined as the correlation between protein expression profile and the module eigengene, was measured with values in range of 0 and 1; where 0 represents that a gene is not part of the module while 1 represents high connectivity to the module. Further, to identify clinically relevant modules related to a specific cancer type, module-trait association was determined using correlation between the module eigengene and lung cancer subtypes (trait). For each protein, a gene significance measure (*GS*) between the expression profile and trait was estimated that allows easy identification of proteins strongly associated with a clinical trait. WGCNA analysis was performed using WGCNA R-package [15], implemented as a gadget in Garuda Platform (The Systems Biology Institute, Tokyo, Japan).

### PPI network construction and functional enrichment

STRING (The Search Tool for the Retrieval of Interacting Genes/Proteins) [36], HINT (High-quality INTeractomes) [37], and IID (Integrated Interactions Database) [38] are major protein-protein interaction (PPI) databases that integrate PPIs from multiple PPI databases, i.e., BIOGIRD (Biological General Repository for Interaction Datasets) [39], IntAct (IntAct Molecular Interaction Database) [40], etc. STRING and IID use PPIs that are computationally predicted by state-of-the-art algorithms (these algorithms uses gene expression data, genomics context, orthology-based analyses, automated text mining analyses). HINT does not include computationally predicted PPIs. Computationally predicted PPIs dramatically decreases the false negative rate, though they may increase the false positive rate [38].

In this study we utilized STRING which integrates PPIs obtained from multiple databases (IntAct [41], Reactome [42], DIP (Database of Interacting Proteins) [43], BioGRID [39], MINT (The Molecular INTeraction Database) [44], KEGG (the Kyoto Encyclopedia of Genes and Genomes) [45], NCI/Nature PID (National Cancer Institute—Nature Pathway Interaction Database) [46], The Interactive Fly [47], and BioCyc [48]) and PPIs computationally predicted by several state of the art algorithms that use gene expression data, genomics context, orthology-based analyses, and automated text mining analyses. PPI network analysis was performed for eigengenes in a selected module with STRING database, version 10.5 [36]. Here, the nodes are proteins/genes, which number relies on the number of eigengenes in a module, and edges are the predicted functional associations that are retrieved from KEGG GO databases (http://www.genome.jp/kegg/) and primary literature. The STRING network interaction scores for each node were expressed as a joint probability derived from curated databases of experimental results, text mining, and computationally predicted by genetic proximity. STRING networks were calculated under the criteria for linkage only with experiments, databases, textmining, and co-expression with the default settings i.e., medium confidence score: 0.400, network depth: 0 and up to 50 interactions. Functional enrichment results were obtained for canonical pathways under *p* < 0.05.

### Functional enrichment analysis

A hub gene is defined as an abbreviation of a “highly connected gene.” The genes inside co-expression modules have high connectivity and the genes within the same module may play similar roles. The PPI networks were reconstructed by the software Cytoscape version 3.6.0., followed by importing of results obtained from the STRING PPI network analysis of eigengenes in each module. We identified hub genes in each module according to their intra-modular connectivity and correlation with module eigengenes. The top 20 high-degree genes were identified by using the *cytoHubba* plugin [49]. The 3 top ranked genes in every module were considered to be hub genes.

## Results and discussion

Both SCLC and LCNEC are malignant and show poor prognosis compared with NSCLC; new molecular information for biological characteristics of SCLC and LCNEC may provide more effective therapeutic strategy. The primary objectives of this study were to capture molecular insights into the tumorigenic difference between SCLC and LCNEC and to construct a gene co-expression network using WGCNA to identify and/or predict the candidate key network modules and Hub genes characteristically associated with the carcinogenesis of each cancer subtype.

### Proteome datasets of SCLC and LCNEC

A total of 974 cases of lung cancer underwent surgery in the period from 2000 to 2014 at the St. Marianna University Hospital. Overall, 41 cases (4.2%) were histologically evaluated as neuroendocrine tumors. Among the total 41 cases, twelve FFPE tissue specimens (SCLC, six specimens; LCNEC, six specimens) were selected especially based on their preserved condition, tumor area, and well-clarified pathology diagnosis (Table 1). No pre-surgical treatment was performed in any of the cases.

**Table 1:** Clinicopathological information of patients.

Sample No.	Histological Type	Age	Sex	Location	Tumor size on CT (mm)	Clinical TNM classification*			Clinical stage
						c-T	c-N	c-M	
A. Small-cell lung cancer (SCLC) (*n* = 6)									
SCLC1	Combined SCLC (SCLC and AD)	74	M	RS1	23	cT1b	cN0	cM0	cIA
SCLC2	SCLC	59	F	RS6	26	cT1b	cN0	cM0	cIA
SCLC3	SCLC	77	M	RS2	12	cT1a	cN0	cM0	cIA
SCLC4	Combined SCLC (SCLC and AD)	64	M	RS3	32	cT2a	cN0	cM0	cIB
SCLC5	Combined SCLC (SCLC and AD)	68	M	RS9	16	cT1a	cN0	cM0	cIA
SCLC6	SCLC	76	M	RS2	19	cT1a	cN0	cM0	cIA
	Average ± SD	70 ± 7	M(83.3%) F(16.7%)		21± 7				
B. Large-cell neuroendocrine lung cancer (LCNEC) (*n* = 6)									
LCNEC1	LCNEC	52	M	RS1	58	cT3	cN0	cM0	cIIB
LCNEC2	LCNEC	79	M	LS4	33	cT2a	cN0	cM0	cIB
LCNEC3	LCNEC	55	M	RS10	19	cT1a	cN0	cM0	cIA
LCNEC4	LCNEC	77	M	RS1	33	cT2a	cN0	cM0	cIB
LCNEC5	LCNEC	66	M	RS3	33	cT2a	cN2	cM0	cIIIA
LCNEC6	LCNEC	69	M	RS2	19	cT1a	cN0	cM0	cIA
	Average ± SD	66 ± 11	M(100%) F(0%)		33 ± 14				
Group comparison	*p*-value (t-test)	0.551			0.118				

*Note*: AD, Adenocarcinoma;

*Staging was determined according to IASLC criteria edition 7th.

**Fig 1:**
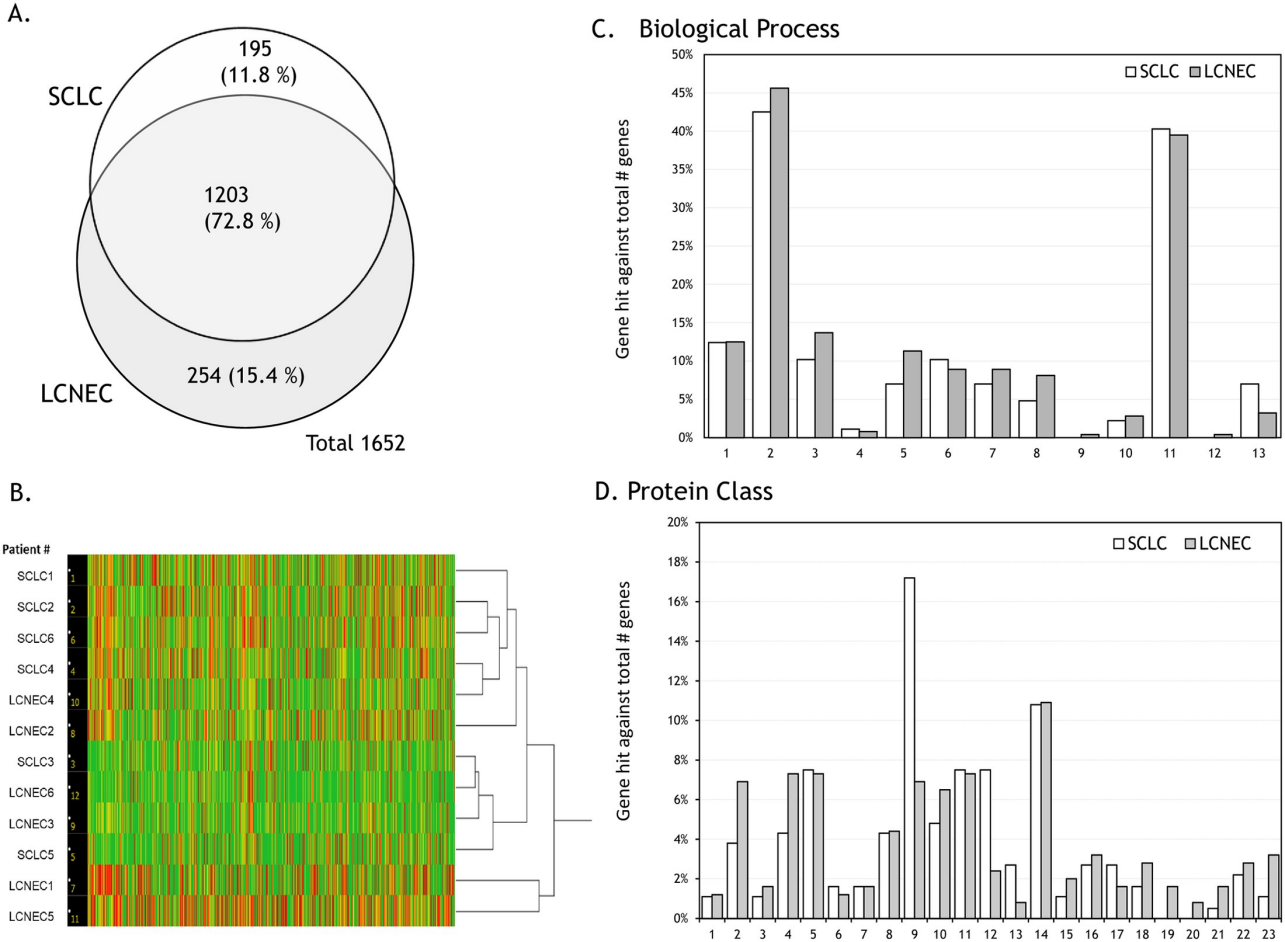
A Venn map, hierarchical clustering, and gene ontology (GO) analysis of the identified proteins. A. Venn map of identified proteins. B. A hierarchical clustering of the expressed proteins using the Ward method including their spectral counts for patients. C. Biological processes: 1, cellular component organization or biogenesis (GO:0071840); 2, cellular process (GO:0009987); 3, localization (GO:0051179); 4, reproduction (GO:0000003);5, biological regulation (GO:0065007); 6, response to stimulus (GO:0050896); 7, developmental process (GO:0032502); 8, multicellular organismal process (GO:0032501); 9, locomotion (GO:0040011); 10, biological adhesion (GO:0022610); 11, metabolic process (GO:0008152);12, growth (GO:0040007); 13,immune system process (GO:0002376). D. Protein classes: 1, extracellular matrix protein (PC00102); 2, cytoskeletal protein (PC00085); 3, transporter (PC00227); 4, transferase (PC00220); 5, oxidoreductase (PC00176); 6, lyase (PC00144); 7, cell adhesion molecule (PC00069); 8, ligase (PC00142); 9, nucleic acid binding (PC00171); 10, signaling molecule (PC00207); 11, enzyme modulator (PC00095); 12, calcium-binding protein (PC00060); 13, defense/immunity protein (PC00090); 14, hydrolase (PC00121); 15, transfer /carrier protein (PC00219); 16, membrane traffic protein (PC00150); 17, transcription factor (PC00218); 18, chaperone (PC00072); 19, cell junction protein (PC00070); 20, surfactant (PC00212); 21, structural protein (PC00211); 22, isomerase (PC00135); 23, receptor (PC00197).

A total of 1,652 proteins were identified among which 1,203 proteins (72.8%) were commonly expressed in both histological types, 195 proteins (11.8%) were unique to SCLC, and 254 proteins (15.4%) were found only in LCNEC. The mass spectrometry data have been deposited to the PRIDE Archive (http://www.ebi.ac.uk/pride/archive/) via the PRIDE partner repository and jPOST with the data set identifier PXD013583 and JPST000587, respectively. Protein expression data obtained from FFPE clinical tissue specimens obtained by surgical resection from 12 patients are provided in S1 File. This demonstrated that both SCLC and LCNEC share the majority of expressed proteins (Fig 1A). A hierarchical clustering was obtained by using the Ward method for proteins with their spectral counts identified for each patient. This did not show a clear separation but a somewhat mixed feature between SCLC and LCNEC (Fig 1B). The patient dendrogram in Fig 1B suggested a pairwise similarity in the protein expression profiles obtained for respective four pairs of patients, which are SCLC2 and SCLC6, SCLC4 and LCNEC4, SCLC3 and LCNEC6, and LCNEC1 and LCNEC5. The former two pairs seem to be closer each other but distant from the latter two pairs. The latter two pairs seem to belong to separated clusters each other. GO analysis using PANTHER Ver. 11.0 [35] exhibited mostly similar profiles in gene hits. However, characteristic differences between SCLC and LCNEC were seen especially in protein class: immune system process, nucleic acid binding, cytoskeletal protein, transferase, calcium-binding protein, defense/immunity protein, chaperone, cell junction protein, surfactant, structural protein, and receptor and so on (Fig 1C and 1D). The biggest difference might be found for cell junction protein and surfactant. 1,203 proteins commonly expressed to both SCLC and LCNEC shared molecular functional properties in binding (40.3%), catalytic activity (31.0%), structural molecule activity (8.2%), transporter activity (3.5%), transcription regulator activity (2.6%), molecular function regulator (2.6%) (S1 Fig). Results of gene set enrichment analysis (GSEA) for GO gene sets performed by using MSigDB (The Molecular Signatures Database: http://software.broadinstitute.org/gsea/msigdb/annotate.jsp) included GO Regulation Of Cell Activation (*p* = 1.14 x 10^−5^) and GO Response To External Stimulus (*p* = 1.19 x 10^−5^) (S1 Table).

### Weighted gene co-expression network identification of modules

**Fig 2:**
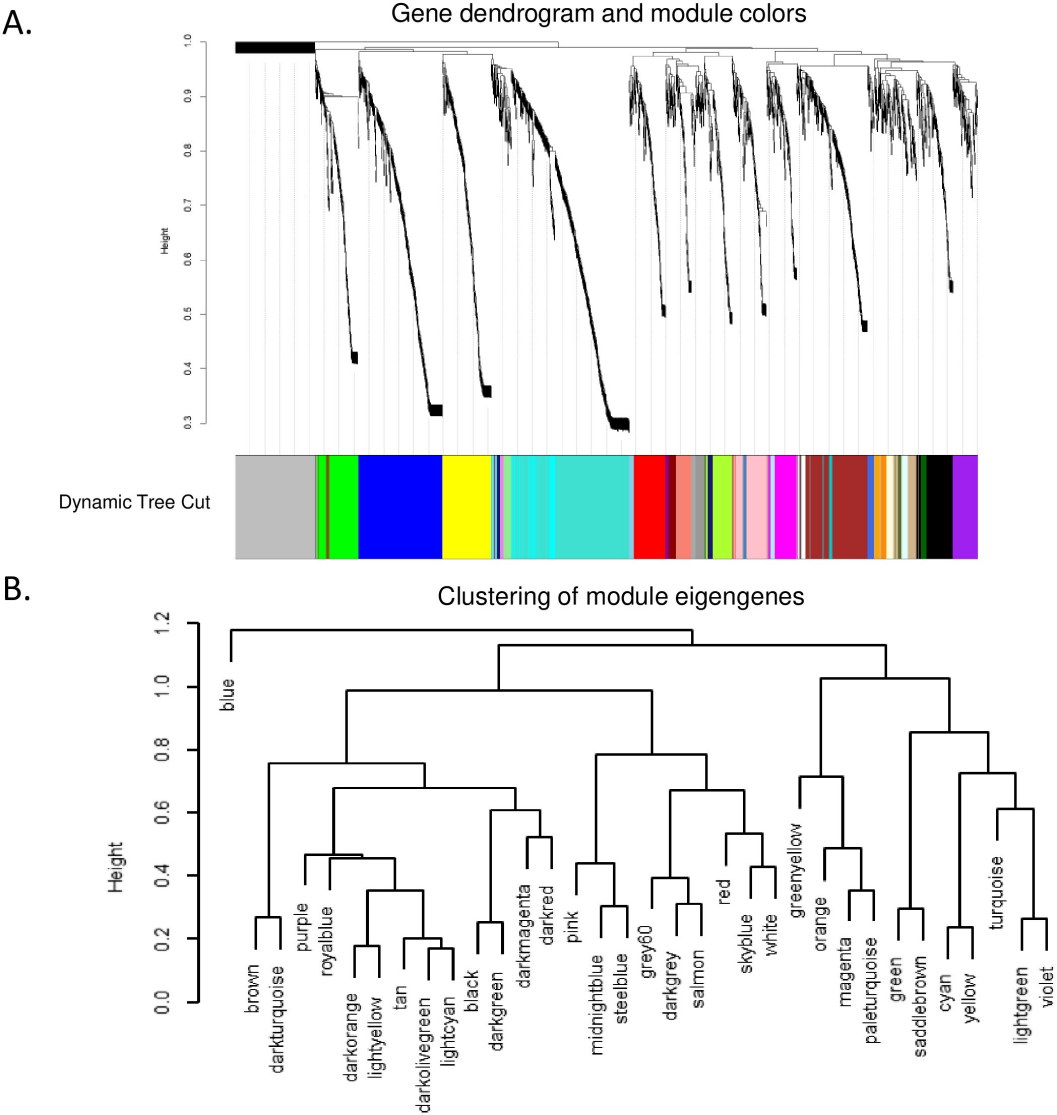
Gene modules identified by weighted gene co-expression network analysis (WGCNA). A. Gene dendrogram obtained by clustering the dissimilarity based on consensus Topological Overlap with the corresponding module. Colored rows respectively correspond 34 modules identified. B. Dendrogram of consensus module eigengenes obtained on the consensus correlation.

A weighted gene co-expression network was constructed using the 1,652 proteins identified with their spectral counts for SCLC and LCNEC patients, and 34 modules were identified by setting SCLC and LCNEC as two traits. The soft threshold power of 10 was chosen to define the adjacency matrix based on the criteria of approximate scale-free topology, with minimum module size 10, the module detection sensitivity *deepSplit* 4, and cut height for merging of modules 0.2. This suggests that the eigengenes in the modules that are correlated above 0.8 would be merged (Fig 2A).

### Correlation between each module

**Fig 3:**
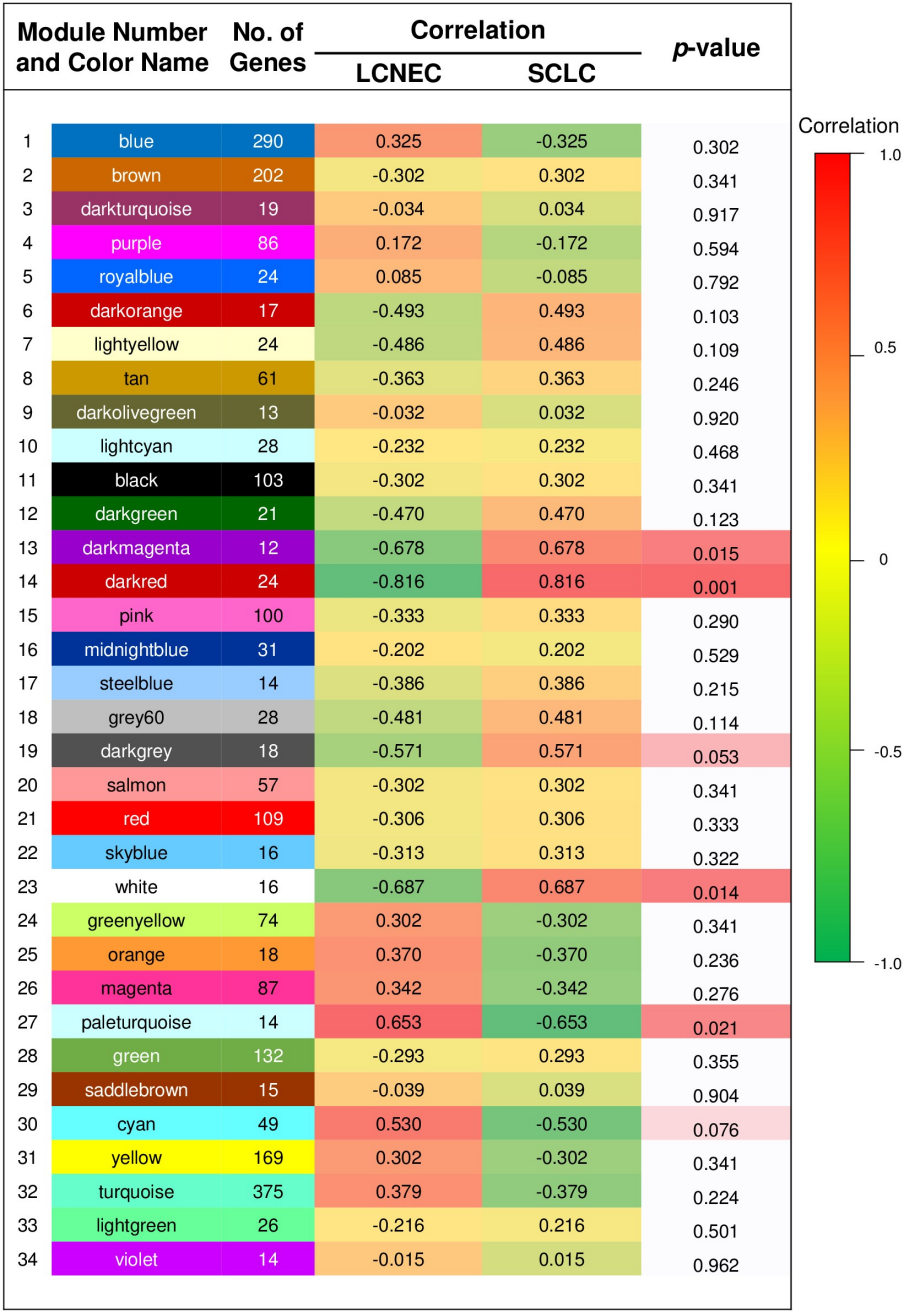
Relationship between consensus module eigengenes and lung neuroendocrine carcinoma subtypes. The first column in the embedded table represents consensus modules, the second column represents the number of eigengenes in each module, the third column indicates the correlations between corresponding module eigengenes to the two lung cancer subtypes (trait), and the last column represents *p*-values. The module with number and color name is shown on the left side of each cell. The table is color coded by correlation according to the color legend. Intensity and direction of correlations are indicated on the right side of the heatmap (red, positively correlation; green, negatively correlation).

As shown in Fig 2B, a cluster analysis on the connectivity of eigengenes was performed within the interactions among the 34 co-expressed modules. Interestingly, the modules were grouped into two clusters, a larger and a smaller. While the smaller cluster contained only one module, multiple sub-clusters were observed in the large cluster with each containing two branches. Combined with Fig 3, a significant difference was observed among the 34 modules. However, no pair of modules below the threshold (0.2) was merged and multiple modules related to SCLC and/or LCNEC subtypes were observed.

**Fig 4:**
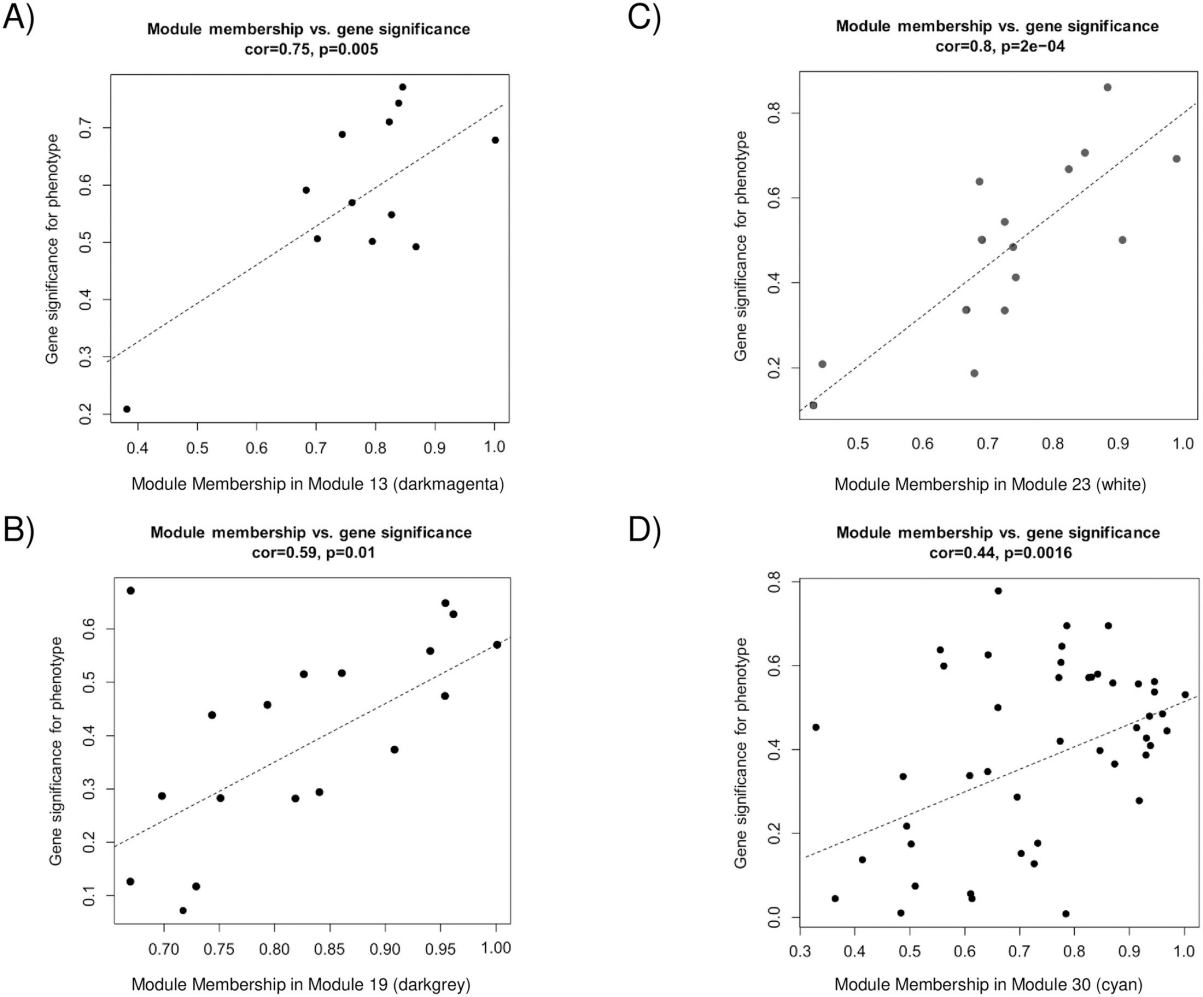
Relationship between gene significance and module membership. Gene significances are plotted against module memberships for the modules of A) 13 (darkmagenta), B) 19 (darkgray), C) 23 (white), and D) 30 (cyan).

A gene significance measure, as a function *GS*, assigns a non-negative number to each gene. The higher the *GS*_*i*_, the more biologically or clinically significant is the gene ‘*i’*. A gene significance measure suggests pathway membership on functional enrichment. Modules with high trait significance suggest pathways associated with the lung cancer subtype sample (trait). Genes with a high module membership (MM) within modules related to the lung cancer subtypes (traits) can be obtained from a correlation between gene significance and module membership. The eigengenes in each module are listed in S2 Table. The modules of 13 (darkmagenta), 19 (darkgray), 23 (white), and 30 (cyan) showed good correlations (Fig 4). Genes with high MM were considered as candidates for further validation [50, 51].

### Functional enrichment analysis

**Fig 5:**
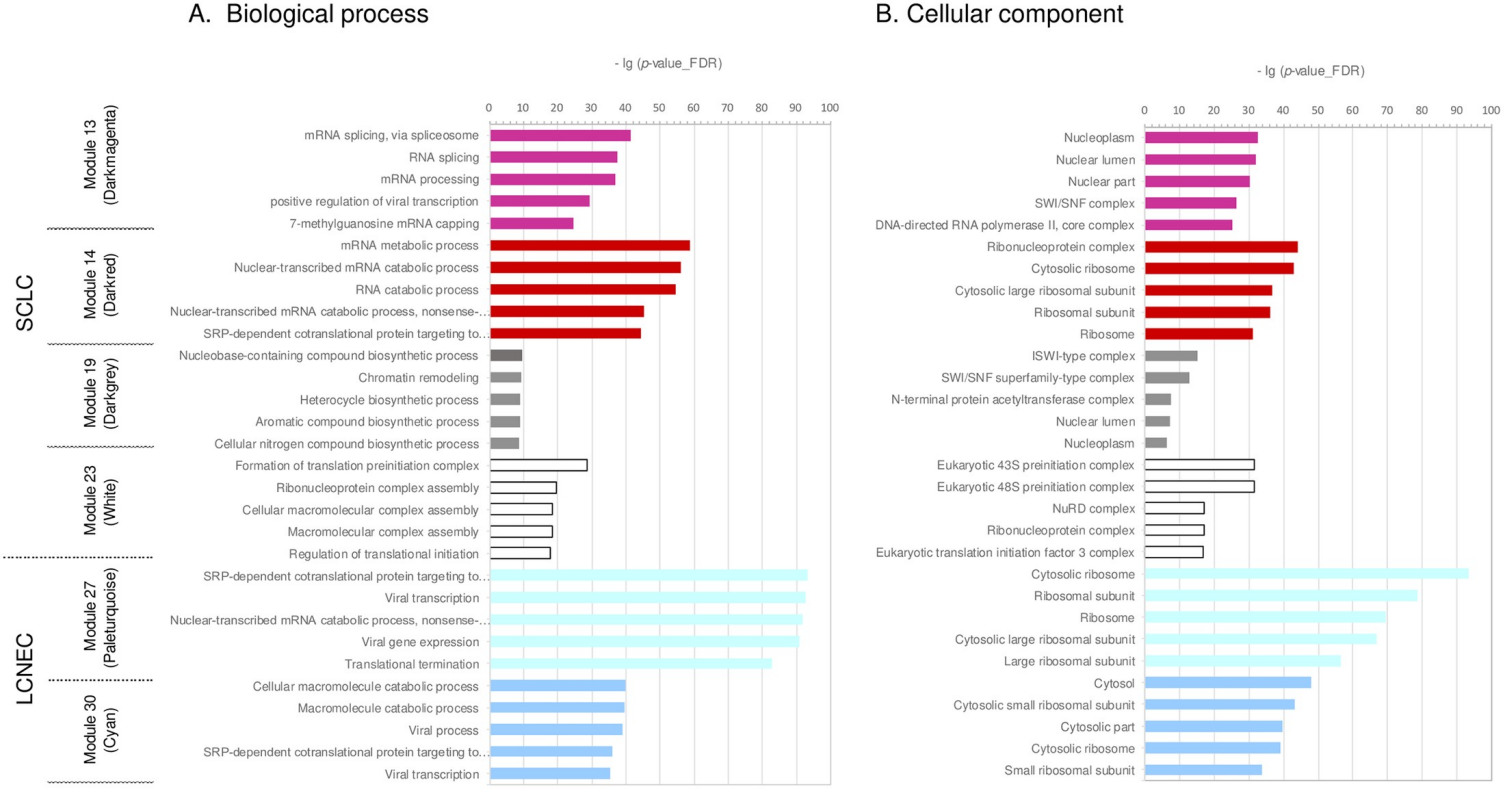
Pathway analysis and top five enrichment results. Enrichment analysis were performed for A. biological processes and B. cellular components by the STRING database for co-expressed genes in the modules of 13 (darkmagenta), 14 (darkred), 19 (darkgray), 23 (white), 27 (paleturquoise), and 30 (cyan). The names of pathways are shown on the left, and the bars on the right represent the −lg (*p*-value_FDR) of the corresponding pathway. The different colors of the bars are in accordance with the corresponding modules.

Six modules were found to be intimately associated with the two lung NET subtypes. Among these modules, 13 (darkmagenta), 14 (darkred), 19 (darkgray), and 23 (white) were significantly associated with SCLC while the modules of 27 (paleturquoise) and 30 (cyan) with LCNEC. These six modules were selected as input for the STRING database network analysis [36]. Fig 5 summarizes the top 5 results of gene ontology (GO) enrichment for pathway analysis on biological processes and cellular components by the STRING PPI network for co-expressed genes. The PPI network information (number of nodes, number of edges, average node degree, PPI enrichment *p*-value etc.) performed for pathway enrichment are provided in S3 Table.

The enriched pathways in Module 13 (darkmagenta) involved DNA transcription processes, including alternative mRNA processing pathways. Those processes particularly involved where DNA-directed RNA polymerase II (RNAP II and Pol II) core complex is activated. The SWI/SNF complex was also enriched, which suggested its participation in active gene transcription regulation. The mRNA metabolic process, especially the nuclear-transcribed mRNA catabolic process of nonsense-mediated decay (NMD), was enriched in Module 14 (darkred). Representative pathways enriched in Module 19 (darkgray) were both nucleobase-containing compound biosynthetic processes and chromatin remodeling comprising of the Imitation SWItch (ISWI)-type and SWI/SNF superfamily-type complexes. This might imply an involvement of both transcription-coupled nucleotide excision repair and DNA double strand break (DBS), via nucleosome remodeling. Enriched pathways in Module 23 (white) included mainly translation initiation processes mediated by the eukaryotic translation initiation factor 43S/48S 3 (eIF3), which plays a central role in the initial recruitment of the preinitiation complex (PIC) onto mRNAs. Simultaneously, the pathways of alternative mRNA splicing and NuRD complex were actively involved in Module 23 (white). NuRD is one of four major ATP-dependent chromatin remodeling complexes and has numerous effects on gene activation and transcription, vesicle-mediated transport-related transport protein particle complex, and cell-cycle-related networks including PCNA and MCM2. The entire spectrum of network relations in Module 23 (white) indicated a highly activated transcriptional activity, protein translation and their vesicular transport. All of these activities are also associated with enhanced DNA damage repair (DDR) activity and cell-cycle turnover.

The common enriched pathways in the modules of 27 (paleturquoise), and 30 (cyan) were signal recognition particle (SRP)-dependent co-translational protein targeting. The SRP is a key component of the cellular machinery that couples the ongoing synthesis of proteins to their proper localization. This is particularly critical for the targeting of integral membrane proteins, which is controlled by the endoplasmic reticulum. The ribosomal proteins of the cytosolic large subunit 60S and the cytosolic small subunit 40S were involved in Module 27 (paleturquoise), and Module 30 (cyan), respectively.

We thus observed that the DNA transcription processes mediated by DNA-directed Pol II core complex, nucleotide excision repair and DNA DBS via nucleosome remodeling, mRNA metabolic processes including mRNA catabolic process NMD, and translation initiation processes mediated by eIF3 were highly activated in SCLC. The co-translational targeting of proteins by SRP was found to be differentially activated in LCNEC. Recently, the novel unsupervised deep learning approach namely, Sample Learning based on Deep Sparse Filtering (SLDSF) [52], was applied for the selection of genes characteristic to the lung cancer dataset including 12,600 genes from 203 lung cancer tissue samples (Bhattacharjee et al. [53]). Nuclear-transcribed mRNA catabolic processes, nonsense-mediated decay, SRP-dependent co-translational protein targeting to membrane, and translational termination, all of which are closely related to lung cancer, were reported as highly significant GO terms (top 10 *p*-values) corresponding to the selected characteristic genes. Thus, our findings are consistent with previous results, in a method-independent manner.

The six modules selected in our study may be categorized globally as processes relevant to gene transcription regulation, alternative mRNA splicing, translational initiation, and protein translocation, which are crucial events in genome integrity and cell-cycle progression, and wherein the major chromatin remodeling complexes also play important roles. It may therefore be suggested that a high DDR and NMD activity most likely occur in both SCLC and LCNEC. The disease-related key network modules identified in this study potentially reflects the deregulation of translational control, thereby inducing rapid and dramatic translational reprogramming both by increasing the overall protein synthesis and by modulating specific mRNA networks. This appears to be the common mechanism via which the diverse oncogenic pathways promote cellular transformation and tumor development.

### Master and upstream regulators predicted by IPA

The investigation of clinically significant modules and their upstream regulators, which play key roles in lung cancer subtypes, was one of the primary reasons for performing co-expression analysis. Analysis of master and upstream regulators was performed for the genes from selected modules using causal network analysis by the Ingenuity Pathway Analysis (IPA, http://www.ingenuity.com) software [54]. As shown in Table 2, several upstream regulators were predicted and included transcriptional regulators, transporters, microRNAs, growth factors and enzymes, etc. Master genes predicted for the modules significant to SCLC included *MADD*, *VEGFC*, *SYVN1*, *ELP3*, *VCAM1*, *IRF7*, *TGFBR*, *CRK*, *CDKN1A*, etc. Those that were significant to LCNEC included *PLA2G6*, *PDPK1*, *FCGR2A*, *TNFAIP8L2*, *TRAP1*, *MXI1*, *TRAF2*, etc.

**Table 2:** Top 5 master regulators of selected modules predicted by causal network analysis using ingenuity pathway analysis (IPA).

Module	Master Regulator	Molecule Type	Participating regulators	Depth	Network bias-corrected p-value	Target molecules in dataset
Module 13 (darkmagenta)	*CTU1*	other	*CTU1*	1	0.0024	*DEK*
	*ELP3*	enzyme	*ELP3*	1	0.0024	*DEK*
	*mir-489*	microRNA	*mir-489*	1	0.0031	*DEK*
	*CD40LG*	cytokine	*CD40LG*	1	0.0076	*CSTF2*
	*Gm15807/Hmgn5*	other	*Gm15807/Hmgn5*	1	0.0098	*HNRNPK*
Module 14 (darkred)	*Fibrinogen*	complex	*CASP8*,*CBL*,*CHUK*,*EGFR*,*Fibrinogen*,*HIF1A*,*IKBKB*,*ITGB3*,*MAPK3*,*MAPK9*,*MTOR*,*NFE2L2*,*PGR*,*RELA*,*SHC1*,*SRC*,*TP53*	3	0.0001	*COPS5*,*CSE1L*,*EIF4E*,*FUBP1*,*HADH*,*HNRNPD*,*LSM3*,*MARCKSL1*,*MAT2A*,*PGRMC1*,*RALY*,*RPL18*,*TXN*
	*PELI3*	enzyme	*CASP8*,*CHUK*,*EGFR*,*HIF1A*,*IKBKB*,*IKK (complex)*,*Jnk*,*MAP3K7*,*MAPK9*,*MTOR*,*NFE2L2*,*NFkB (complex)*,*NFKB1*,*NFKBIA*,*PELI3*,*RELA*,*RICTOR*,*TAB1*,*TP53*	3	0.0001	*COPS5*,*CSE1L*,*EIF4E*,*FUBP1*,*HADH*,*HNRNPD*,*LSM3*,*MARCKSL1*,*MAT2A*,*PGRMC1*,*RALY*,*RPL18*,*TXN*
	*TGFBR*	group	*CASP8*,*EGFR*,*FLI1*,*MAPK9*,*MTOR*,*NFE2L2*,*PRKCD*,*RELA*,*SMAD3*,*TGFBR*,*TP53*	3	0.0001	*COPS5*,*CSE1L*,*EIF4E*,*FUBP1*,*HADH*,*LSM3*,*MARCKSL1*,*MAT2A*,*PGRMC1*,*RALY*,*RPL18*,*TXN*
	*CRK*	other	*CASP8*,*CRK*,*EGFR*,*ERK*,*MAPK1*,*MAPK3*,*MTOR*,*NFE2L2*,*NOS2*,*RAC1*,*RARA*,*RELA*,*SHC1*,*TP53*	3	0.0001	*API5*,*COPS5*,*CSE1L*,*EIF4E*,*FUBP1*,*HADH*,*HNRNPD*,*MAT2A*,*PGRMC1*,*RALY*,*RPL18*,*TXN*
	*CDKN1A*	kinase	*CASP8*,*CDKN1A*,*MAPK9*,*NFE2L2*,*TP53*	2	0.0001	*COPS5*,*CSE1L*,*EIF4E*,*FUBP1*,*HADH*,*LSM3*,*MARCKSL1*,*RALY*,*RPL18*,*TXN*
Module 19 (darkgrey)	*miR-342-3p (miRNAs w/seed CUCACAC)*	mature microRNA	*miR-342-3p (miRNAs w/seed CUCACAC)*	1	0.0013	*MTDH*
	*FTX*	other	*FTX*	1	0.0021	*MTDH*
	*IRF7*	transcription regulator	*IRF7*	1	0.0046	*PARP14*,*UBE2L6*
	*mir-342*	microRNA	*mir-342*	1	0.0049	*MTDH*
	*VCAM1*	transmembrane receptor	*CBL*,*ELK1*,*HIF1A*,*IRF3*,*ITGB1*,*KRAS*,*MAPK1*,*PI3K (complex)*, *PRKCA*, *SPHK1*,*TGFB1*,*TP73*,*VCAM1*	3	0.0054	*ARHGEF2*,*CARS*,*CNN3*,*IGF2BP2*,*PARP14*,*TAGLN*,*TYMP*,*UBE2L6*
Module 23 (white)	*MADD*	other	*ERK*,*MADD*,*MAP2K1/2*	2	0.0014	*CTTN*,*RRM1*
	*COL4A3BP*	kinase	*COL4A3BP*,*ERBB2*,*ERK*	2	0.0028	*CHD4*,*CTTN*,*RRM1*
	*VEGFC*	growth factor	*VEGFC*	1	0.0031	*CTTN*
	*BTRC*	enzyme	*BTRC*	1	0.005	*CTTN*
	*SYVN1*	transporter	*SYVN1*	1	0.0068	*HNRNPM*,*TARS*
Module 27 (paleturquoise)	*PLA2G6*	enzyme	*AKT1*,*AR*,*CEBPA*,*CEBPB*,*ELK1*,*ERBB2*,*Jnk*,*MYC*,*NFE2L2*,*P38 MAPK*, *PLA2G6*,*RICTOR*, *Rock*	3	0.0002	*APCS*,*EDF1*,*HPX*,*LIG3*,*MTHFD2*,*POLDIP3*,*RPL14*,*SNRPD1*
	*PDPK1*	kinase	*AKT1*,*CEBPA*,*CEBPB*,*ERBB2*,*GSK3B*,*IKBKB*,*MYC*,*NFE2L2*,*NFkB (complex)*, *PDPK1*, *PRKCD*,*PRKCG*,*RICTOR*,*RPS6KB1*,*RTN4*,*TNFRSF1A*	3	0.0003	*APCS*,*DPYSL5*,*EDF1*,*HPX*,*LIG3*,*POLDIP3*,*RPL14*,*SNRPD1*
	*FCGR2A*	transmembrane receptor	*CEBPA*,*CEBPB*,*ELK1*,*ERBB2*,*ERK*,*FCGR2A*,*MYC*,*MYD88*,*NFkB (complex)*, *Pkc(s)*, *PTPN6*,*Rac*,*REL*,*SYK*,*TNFRSF1A*	3	0.0004	*ACAD9*,*APCS*,*EDF1*,*HPX*,*LIG3*,*MTHFD2*,*POLDIP3*,*SNRPD1*
	*TNFAIP8L2*	other	*AR*,*CEBPA*,*CEBPB*,*ELK1*,*Jnk*,*MYC*,*NFE2L2*,*P38 MAPK*,*Rac*,*RICTOR*,*TNFAIP8L2*	3	0.0009	*APCS*,*EDF1*,*HPX*,*MTHFD2*,*POLDIP3*,*RPL14*,*SNRPD1*
	*PPM1A*	phosphatase	*AR*,*CDK9*,*CEBPA*,*CEBPB*,*ELK1*,*ERBB2*,*Jnk*,*MAP2K4*,*MYC*,*NFE2L2*,*P38 MAPK*,*PPM1A*,*RICTOR*,*TP53*	3	0.0009	*APCS*,*EDF1*,*HPX*,*LIG3*,*MTHFD2*,*POLDIP3*,*RPL14*,*SNRPD1*
Module 30 (cyan)	*TRAP1*	enzyme	*TRAP1*	1	0.0002	*GARS*,*MAVS*,*PKM*
	*CXCL12*	cytokine	*CXCL12*	1	0.0005	*EXOSC6*,*RANGAP1*,*TF*
	*BTRC*	enzyme	*ATF4*,*BTRC*,*CTNNB1*,*MTOR*,*MTORC1*,*NFkB (complex)*, *RELA*	2	0.0006	*ACLY*,*ACTC1*,*CLTC*,*GARS*,*IARS*,*LAMB1*,*PKM*,*RAB3C*
	*MXI1*	transcription regulator	*MXI1*	1	0.0011	*IARS*,*IMPDH2*
	GPR84	G-protein coupled receptor	GPR84	1	0.0016	PTGES2

Participating regulators are regulators through which the upstream regulator molecule controls the expression of target molecules in dataset. Target molecules in dataset are molecules in our dataset whose expression is potentially controlled by upstream regulator.

All these master genes have important roles in carcinogenesis and tumorigenesis. *MADD* encodes MAP kinase-activating death domain protein which plays a significant role in regulating cell proliferation, survival and death through alternative mRNA splicing, and links *TNFRSF1A* with MAP kinase activation [55]. *VEGFC* encodes vascular endothelial growth factor C protein, which is active in angiogenesis and endothelial cell growth, and stimulates their proliferation and migration [56, 57]. *SYVN1* encodes E3 ubiquitin-protein ligase synoviolin, which suppresses the expression of p53/TP53 in the cytoplasm and promotes its degradation, thereby negatively regulating its biological function in transcription, cell-cycle regulation, and apoptosis [58]. *ELP3* encodes a component of the Pol II holoenzyme and is involved in transcriptional elongation [59]. *CDKN1A* encodes cyclin-dependent kinase inhibitor 1, and is involved in p53/TP53 mediated inhibition of cellular proliferation in response to DNA damage, and inhibits cyclin-dependent kinase activity, preventing phosphorylation of critical cyclin-dependent kinase substrates and blocking cell-cycle progression [60]. *TNFAIP8L2* encodes tumor necrosis factor alpha-induced protein 8-like protein 2 (TIPE2), which is a negative regulator of Toll-like receptor, T-cell receptor function, and also a regulator of the apoptotic process [61]. *TRAP1* encodes tumor necrosis factor type 1 receptor-associated protein, which is a negative regulator of mitochondrial respiration able to modulate the balance between oxidative phosphorylation and aerobic glycolysis (referred to as Warburg effect [62]) [63]. *MXI1* encodes Myc-associated factor X (Max)-interacting protein 1, which is a transcriptional repressor and antagonizes *MYC* transcriptional activity by competing for *MAX* [64]. *TRAF2* encodes TNF receptor-associated factor 2, which regulates activation of NF-kappa-B and JNK and plays a central role in the regulation of cell survival and apoptosis [65]. Most of the functions of the predicted master genes appear as overlaps and are consistent globally with the GO enrichment results of pathway analysis for the selected modules. Identification of master and upstream regulators for the key modules would be useful in aiding the discovery of therapeutic targets.

Nevertheless, the mechanisms underlying the formation of molecular machineries based on these key regulators during tumorigenesis of both SCLC and LCNEC is not clearly understood and will require substantial in-depth investigation in future.

### High-degree genes in PPI networks of selected modules

**Fig 6:**
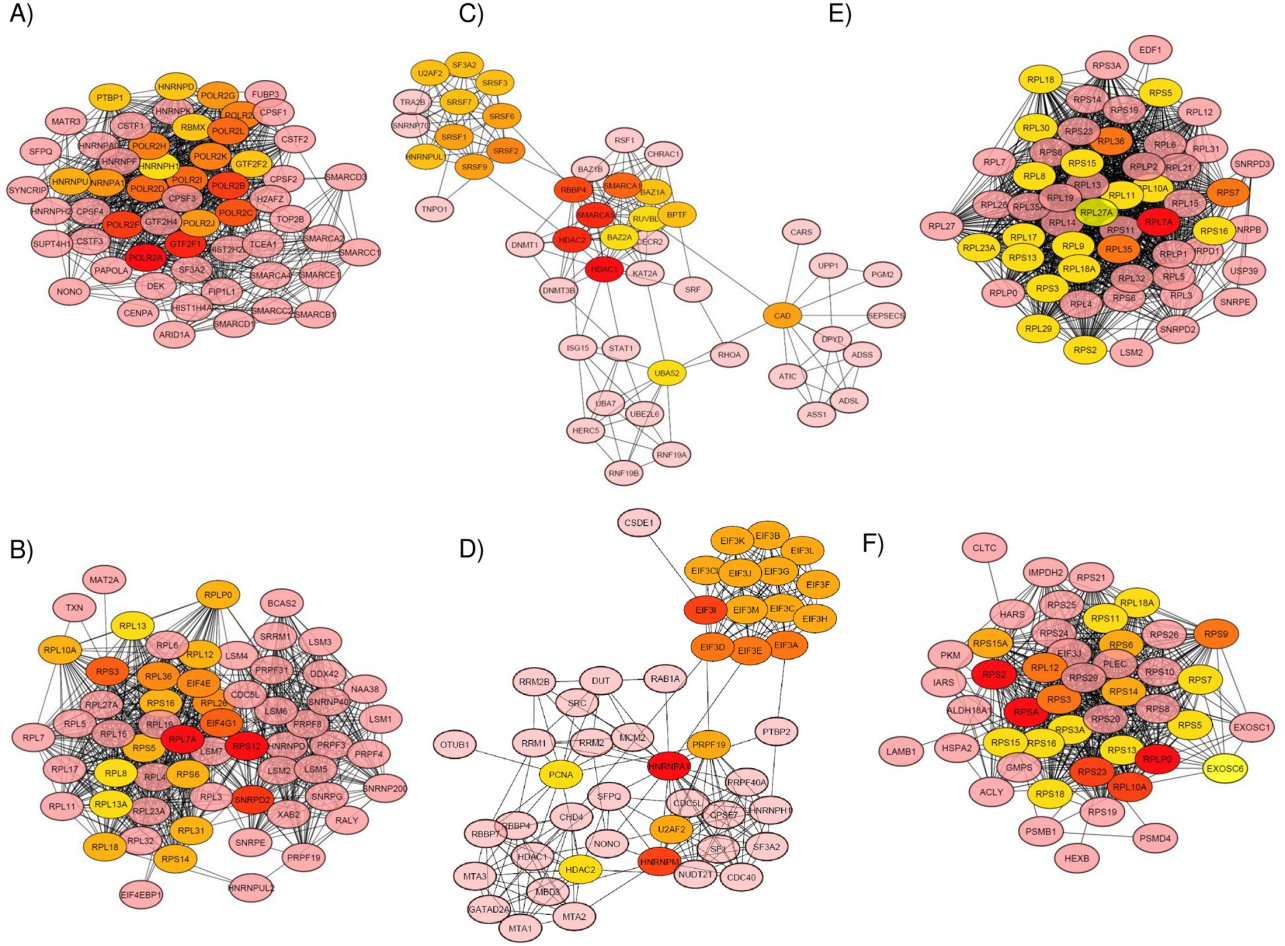
The PPI networks reconstructed by using Cytoscape 3.6 software for the modules. A) 13 (darkmagenta), B) 14 (darkred), C) 19 (darkgray), D) 23 (white), E) 27 (paleturquoise), and F) 30 (cyan). The high-degree genes were calculated by the *cytoHubba* plugin, and the high-degree genes (nodes) are shown with a color scheme from highly essential (red) to essential (green) [49].

High-degree genes are genes (nodes) by their network features to infer their higher importance in the network. To identify high degree genes in the six selected modules (13 (darkmagenta), 14 (darkred), 19 (darkgray), 23 (white), 27 (paleturquoise), and 30 (cyan)), the PPI network was generated using STRING database queried by *cytoHubba* [49] plugin in Cytoscape version 3.6.0 (https://cytoscape.org/) as shown in Fig 6. STRING database is one of the most inclusive of a number of online PPI providing a comprehensive depth in the protein interactome. By combining these data sources, STRING assigns a confidence score to each interaction pair that could be used to filter the false positive interactions. All high-degree genes were calculated by the *cytoHubba* plugin [49], and the high-degree genes (nodes) are shown with a color scheme from highly essential (red) to essential (green). The top 20 genes are listed in S4 Table).

In Module 13 (darkmagenta), the top 3 high-degree genes were *POLR2A*, *GTF2F1* and *POLR2F*, which respectively encode the first and sixth largest subunits of RNAP II/Pol II, the polymerase responsible for synthesizing mRNA in eukaryotes [66]. *GTF2F1/TFIIF* encodes a general transcription initiation factor that binds to RNAP II/Pol II and helps its recruitment onto the initiation complex in collaboration with TFIIB, which promotes transcription elongation [67]. In Module 14 (darkred), *RPS12*, *RPL7A*, and *SNRPD2* were the top 3 high-degree genes. Proteins encoded by these genes belong to the cytosolic 40S small and 60S large ribosomal subunits, respectively, which are involved in mRNA metabolic processes including NMD. NMD is a eukaryotic surveillance mechanism that monitors cytoplasmic mRNA translation and targets premature translation terminated mRNAs for rapid degradation [68]. The protein encoded by *SNRPD2* belongs to the small nuclear ribonucleoprotein core protein family, which is required for pre-mRNA splicing and small nuclear ribonucleoprotein biogenesis in the major mRNA splicing pathway. The top 3 high-degree genes in Module 23 (white) were *HNRNPA1*, *HNRNPM*, and *EIF3I*. *HNRNPA1* and *HNRNPM* are involved in the major mRNA splicing pathways. *EIF3I* encodes a component of the eIF-3 complex, which is required for several steps in the initiation of protein synthesis. In Module 19 (darkgray) they were *HDAC1*, *HDAC2*, and *SMARCA5*, the members of chromatin remodelers. HDAC1 and HDAC2 are the components of the histone deacetylase complex, which interact with the retinoblastoma tumor suppressor protein pRb, which plays a key role in the control of cell proliferation and differentiation. Together with the metastasis-associated protein-2 (MTA2), pRb also deacetylates p53 and modulates its effect on cell growth and apoptosis. *SMARCA5/SNF2H* encodes a member of the ISWI family of chromatin remodelers, which has the ability to remodel chromatin by sliding and displacing nucleosomes, and its accumulation and spreading at DNA lesions of DSBs are triggered with poly(ADP-ribosyl)ation by poly(ADP-ribose) polymerase 1 (PARP1).

In Module 27 (paleturquoise), they were, *RPL7A*, *RPL35*, and *RPL36*, which are involved in SRP-dependent co-translational protein targeting to membrane. In Module 30 (cyan), the top 3 high-degree genes were *RPSA*, *RPS2*, and *RPLP0*, which are involved in cellular macromolecule catabolic processes including SRP-dependent co-translational protein targeting to membrane. *RPL35*, *RPL36* and *RPLP0* encode proteins of the cytosolic large ribosome 60S, whereas both *RPSA* and *RPS2* encode proteins of the cytosolic small ribosome 40S. RPSA is required for a late step in the maturation of the 40S ribosomal subunit, functions as a cell surface receptor for laminin, and may associate with cell fate determination and tissue morphogenesis. Proteins encoded by *RPL7A*, *RPL35*, *RPLP0*, *RPSA*, and *RPS2* are significantly associated with SRP-dependent co-translational protein targeting to membranes [69, 70].

WGCNA analysis could identify key network modules and their eigengenes from proteome datasets obtained from clinical tissue specimens. We should note that key network themselves would be multifunctional and participate in various cancer-related pathways and biological processes. It was indicated that the four key modules identified for SCLC were involved significantly in chromatin remodeling pathways relating to a hyperactivation of both DNA damage repair more than LCNEC. However, the functional roles associated with the two key modules for LCNEC were unclear whereas those might be relevant most likely to protein transport, translocation, and macromolecule localization. Our comparative proteome data also exhibited that many proteins related to DNA damage response/repair, NuRD and SWI/SNF complexes, DNA mismatch repair (DMR), and cell cycle were commonly overexpressed in both SCLC and LCNEC, in which included were CHD4, RBBP4, RBBP7, MTA2, HDAC1, SMARCA4, SMARCA2, TOP2B, MSH2, MSH6, MCM2 to MCM7 etc. with their relatively different expressions. (S2 File).

All together were firstly indicative of a predominant similarity in protein expressions and their PPI networks between SCLC and LCNEC. Next-generation sequencing with the MSK-IMPACT test suggested that LCNEC may express distinct SCLC-like molecular subsets including concomitant loss of *RB1* and *TP53*, and subsets that include *KRAS*, *STK11*, *KEAP1*, or *MAP2K1* (*MEK1*) mutations that can be found in NSCLC [71]. However, it is still unclear why LCNEC cells have oncological aspects different from SCLC and morphological feature resembling NSCLC. Secondly, we observed proteins often reported for NSCLC, and found to be expressed significantly to LCNEC in this study. Those were EML4 (echinoderm microtubule-associated protein-like 4), CRKL (Crk-like protein), and MAPK1/ERK2 (mitogen-activated protein kinase 1 / extracellular signal-regulated kinase), and MAP2K1/MEK1, which was also identified as a member of Module 30 (cyan). EML4 modifies microtubule assembly dynamics, and also interacts with anaplastic lymphoma receptor tyrosine kinase (ALK) known to be involved in the PI3K and NSCLC pathways. In the causal network analysis for LCNEC, *PDPK1*, which encodes 3-phosphoinositide-dependent protein kinase 1, was predicted as the master gene targeting to eight genes including *EDF1* (endothelial differentiation-related factor 1), which are the eigengene members belonging to Module 27 (paleturquoise). Interestingly, it was reported that upregulation of hsa_circ_0004015 (a circular RNA) in NSCLC tissues was associated with poor overall survival of NSCLC patients, and could lead to resistance to gefitinib, and that *PDPK1* as the target gene of miR-1183 participates in circ_0016760/miR-1183/*PDPK1* signaling pathway which might be associated with the tumorigenesis of NSCLC [72].

Results obtained in this study could lead to identification of regulating key genes in the disease. As the next step, we currently proceed with a search for regulator genes/proteins responsible for obtained networks. We would like to discuss focusing on key regulator genes in future study.

## Conclusion

We applied WGCNA to clinical proteomic datasets, for the first time to the best of our knowledge, for exploring molecular networks associated with tumorigenesis that characterize SCLC and LCNEC. Four modules were found to be exclusively associated with SCLC and two with LCNEC; several other modules appeared to be shared by both subtypes of lung cancer. Among the six modules, SCLC was particularly characteristic to the active participation of alternative mRNA splicing and chromatin remodeling related pathways, and LCNEC that of the SRP-dependent co-translational protein targeting to membrane (translocation), respectively. A few master and upstream regulators, which play important roles in cancer progression, were predicted by a causal network analysis. The high expression of several identified novel high-degree hub genes were associated with high risk patient groups. These genes may prove to be prognostic and predictive marker candidates for lung cancer. In addition to the results presented in this study, a further in-depth network-based investigation is required for a clearer understanding of pathways and genes underlying both SCLC and LCNEC.

## Supporting information

S1 Fig The GO analyses of 1,203 proteins commonly expressed to both SCLC and LCNEC. A) GO Molecular function, B) Biological process, and C) Protein class.
(DOC)

S1 Table Results of GO gene set enrichment analysis (GSEA) performed for 1,203 proteins commonly expressed to both SCLC and LCNEC. (DOC)

S2 Table Eigengenes in each module. (DOC)

S3 Table The PPI network information performed for pathway enrichment. (DOCX)

S4 Table Top 20 genes in network ranked by degree method. (DOC)

S1 File Protein expression data. (XLS)

S2 File Proteins expressed characteristically in SCLC and LCNEC. (XLSX)
