# Supplementary material for: Identification of key modules and hub genes for small-cell lung carcinoma and large-cell neuroendocrine lung carcinoma by weighted gene co-expression network analysis of clinical tissue-proteomes
Source: PLoS One. 2019 Jun 5;14(6):e0217105. doi: 10.1371/journal.pone.0217105 (PMC6550379; doi:10.1371/journal.pone.0217105)

**S1 Fig. The GO analyses of 1,203 proteins commonly expressed to both SCLC and LCNEC. A) GO Molecular function, B) Biological process, and C) Protein class.**

A)


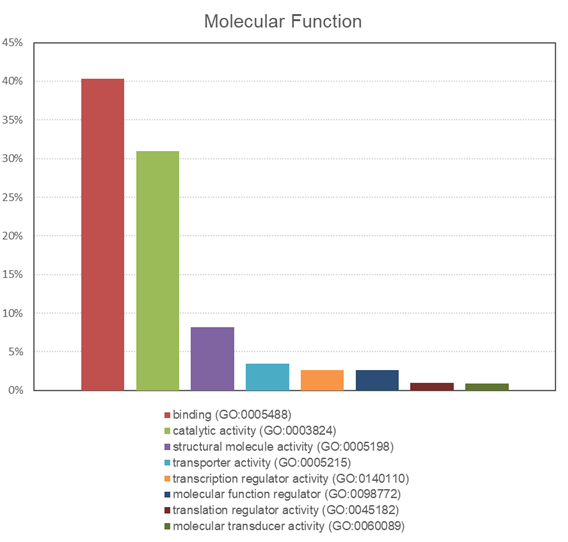


B)


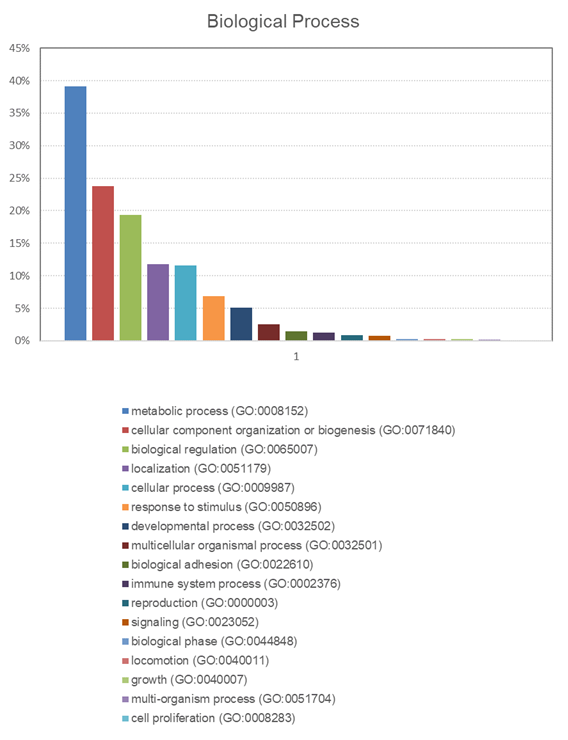


C)


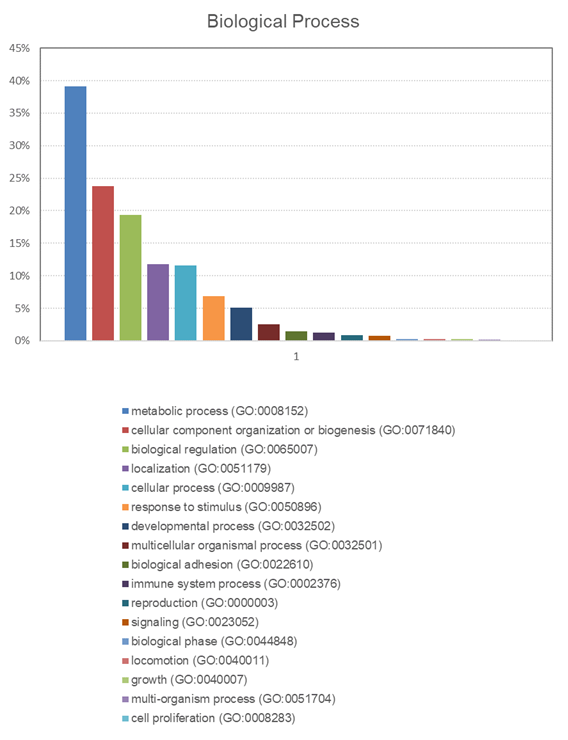

Supplement: S1 Fig — A) GO Molecular function, B) Biological process, and C) Protein class. (DOC) [file pone.0217105.s001.doc]
