# Supplementary material for: Identification of key modules and hub genes for small-cell lung carcinoma and large-cell neuroendocrine lung carcinoma by weighted gene co-expression network analysis of clinical tissue-proteomes
Source: PLoS One. 2019 Jun 5;14(6):e0217105. doi: 10.1371/journal.pone.0217105 (PMC6550379; doi:10.1371/journal.pone.0217105)
Supplement: S1 Table — (DOC) [file pone.0217105.s002.doc]

**S1 Table. Results of GO gene set enrichment analysis (GSEA) performed for**

**1,203 proteins commonly expressed to both SCLC and LCNEC.**

1. Compute Overlaps for Selected Genes

| Collection(s): | C5 |
| --- | --- |
| # overlaps shown: | 10 |
| # genesets in collections: | 5917 |
| # genes in comparison (n): | 5 |
| # genes in universe (N): | 45956 |

| Gene Set Name [# Genes (K)] | # Genes in Gene Set (K) | Description | # Genes in Overlap (k) | k/K | *p*-value | FDR *q*-value |
| --- | --- | --- | --- | --- | --- | --- |
| GO_IMMUNOGLOBULIN_COMPLEX | 26 | A protein complex that in its canonical form is composed of two identical immunoglobulin heavy chains and two identical immunoglobulin light chains, held together by disulfide bonds and sometimes complexed with additional proteins. An immunoglobulin complex may be embedded in the plasma membrane or present in the extracellular space, in mucosal areas or other tissues, or circulating in the blood or lymph. | 2 | 0.0769 | 3.07E-06 | 7.82E-03 |
| GO_IMMUNOGLOBULIN_RECEPTOR_BINDING | 27 | Interacting selectively and non-covalently with one or more specific sites on an immunoglobulin receptor molecule. | 2 | 0.0741 | 3.32E-06 | 7.82E-03 |
| GO_REGULATION_OF_IMMUNE_SYSTEM_PROCESS | 1403 | Any process that modulates the frequency, rate, or extent of an immune system process. | 4 | 0.0029 | 4.22E-06 | 7.82E-03 |
| GO_PHAGOCYTOSIS_RECOGNITION | 34 | The initial step in phagocytosis involving adhesion to bacteria, immune complexes and other particulate matter, or an apoptotic cell and based on recognition of factors such as bacterial cell wall components, opsonins like complement and antibody or protein receptors and lipids like phosphatidyl serine, and leading to intracellular signaling in the phagocytosing cell. | 2 | 0.0588 | 5.31E-06 | 7.82E-03 |
| GO_PHAGOCYTOSIS_ENGULFMENT | 38 | The internalization of bacteria, immune complexes and other particulate matter or of an apoptotic cell by phagocytosis, including the membrane and cytoskeletal processes required, which involves one of three mechanisms: zippering of pseudopods around a target via repeated receptor-ligand interactions, sinking of the target directly into plasma membrane of the phagocytosing cell, or induced uptake via an enhanced membrane ruffling of the phagocytosing cell similar to macropinocytosis. | 2 | 0.0526 | 6.65E-06 | 7.82E-03 |
| GO_POSITIVE_REGULATION_OF_NEURON_APOPTOTIC_PROCESS | 47 | Any process that activates or increases the frequency, rate or extent of cell death of neurons by apoptotic process. | 2 | 0.0426 | 1.02E-05 | 7.82E-03 |
| GO_MEMBRANE_INVAGINATION | 48 | The infolding of a membrane, resulting in formation of a vesicle. | 2 | 0.0417 | 1.07E-05 | 7.82E-03 |
| GO_REGULATION_OF_CELL_ACTIVATION | 484 | Any process that modulates the frequency, rate or extent of cell activation, the change in the morphology or behavior of a cell resulting from exposure to an activating factor such as a cellular or soluble ligand. | 3 | 0.0062 | 1.14E-05 | 7.82E-03 |
| GO_RESPONSE_TO_EXTERNAL_STIMULUS | 1821 | Any process that results in a change in state or activity of a cell or an organism (in terms of movement, secretion, enzyme production, gene expression, etc.) as a result of an external stimulus. | 4 | 0.0022 | 1.19E-05 | 7.82E-03 |
| GO_B_CELL_RECEPTOR_SIGNALING_PATHWAY | 54 | A series of molecular signals initiated by the cross-linking of an antigen receptor on a B cell. | 2 | 0.037 | 1.35E-05 | 8.00E-03 |

B. Gene/geneset overlap matrix.
