# Supplementary material for: Identification of key modules and hub genes for small-cell lung carcinoma and large-cell neuroendocrine lung carcinoma by weighted gene co-expression network analysis of clinical tissue-proteomes
Source: PLoS One. 2019 Jun 5;14(6):e0217105. doi: 10.1371/journal.pone.0217105 (PMC6550379; doi:10.1371/journal.pone.0217105)
Supplement: S2 Table — (DOC) [file pone.0217105.s003.doc]

**S2 Table. Eigengenes in each module.**

| Eigengene No. | 13 (darkmagenta) | 14 (darkred) | 19 (darkgrey) | 23 (white) | 27 (paleturquoise) | 30 (cyan) |
| --- | --- | --- | --- | --- | --- | --- |
| 1 | CSTF2 | API5 | ADSS | CHD4 | ACAD9 | ACLY |
| 2 | DEK | COPS5 | ARHGEF2 | CPSF7 | APCS | ACTC1 |
| 3 | FUBP3 | CSE1L | ASNA1 | CSDE1 | DPYSL5 | ACTG1 |
| 4 | HIST1H4A | DDX42 | CARS | CTTN | EDF1 | ADSL |
| 5 | HNRNPK | EIF4E | CNN3 | EIF3L | HPX | AGL |
| 6 | HNRNPU | FUBP1 | IGF2BP2 | HNRNPM | LIG3 | AGR3 |
| 7 | MATR3 | HADH | MTDH | IPO7 | MTHFD2 | ALDH18A1 |
| 8 | NONO | HNRNPD | NAA15 | IRF2BPL | POLDIP3 | ANXA7 |
| 9 | NUMA1 | HNRNPUL2 | PARP14 | NCOA5 | RPL14 | AP2B1 |
| 10 | RBMX | LSM3 | PGM2 | OTUB1 | RPL29 | ARCN1 |
| 11 | SMARCE1 | MARCKSL1 | SEPHS1 | PTBP2 | RPLP1 | CKMT1A |
| 12 | TCEA1 | MAT2A | SMARCA5 | RRM1 | SNRPD1 | CLTC |
| 13 |  | MAT2B | SRSF9 | SF1 | USP39 | COPA |
| 14 |  | PFDN2 | TAGLN | SFPQ | ZFR | CPSF2 |
| 15 |  | PGRMC1 | TNKS1BP1 | TARS |  | DNAJC7 |
| 16 |  | PRPF19 | TNPO1 | TRAPPC3 |  | EIF3J |
| 17 |  | PRPF3 | TYMP |  |  | EXOSC6 |
| 18 |  | PRPF8 | UBE2L6 |  |  | GARS |
| 19 |  | RALY |  |  |  | GOLM1 |
| 20 |  | RPL18 |  |  |  | HARS |
| 21 |  | SNRNP40 |  |  |  | HEXB |
| 22 |  | SRRM1 |  |  |  | HSPA2 |
| 23 |  | THRAP3 |  |  |  | HSPG2 |
| 24 |  | TXN |  |  |  | IARS |
| 25 |  |  |  |  |  | IMPDH2 |
| 26 |  |  |  |  |  | LAMB1 |
| 27 |  |  |  |  |  | MAP2K1 |
| 28 |  |  |  |  |  | MAVS |
| 29 |  |  |  |  |  | NAE1 |
| 30 |  |  |  |  |  | PICK1 |
| 31 |  |  |  |  |  | PKM |
| 32 |  |  |  |  |  | PLEC |
| 33 |  |  |  |  |  | PPT1 |
| 34 |  |  |  |  |  | PRRC1 |
| 35 |  |  |  |  |  | PSMD8 |
| 36 |  |  |  |  |  | PTGES2 |
| 37 |  |  |  |  |  | RAB3A |
| 38 |  |  |  |  |  | RAB3C |
| 39 |  |  |  |  |  | RAB3D |
| 40 |  |  |  |  |  | RANBP3 |
| 41 |  |  |  |  |  | RANGAP1 |
| 42 |  |  |  |  |  | RPL18A |
| 43 |  |  |  |  |  | RPS15A |
| 44 |  |  |  |  |  | SEC23A |
| 45 |  |  |  |  |  | ST13 |
| 46 |  |  |  |  |  | STARD10 |
| 47 |  |  |  |  |  | STXBP2 |
| 48 |  |  |  |  |  | TF |
| 49 |  |  |  |  |  | VPS25 |
|  |  |  |  |  |  |  |
