# Supplementary material for: Identification of key modules and hub genes for small-cell lung carcinoma and large-cell neuroendocrine lung carcinoma by weighted gene co-expression network analysis of clinical tissue-proteomes
Source: PLoS One. 2019 Jun 5;14(6):e0217105. doi: 10.1371/journal.pone.0217105 (PMC6550379; doi:10.1371/journal.pone.0217105)
Supplement: S3 Table — (DOCX) [file pone.0217105.s004.docx]

**S3 Table. The PPI network information performed for pathway**

| Module | number of nodes | number of edges | average node degree | avg. local clustering coefficient | expected number of edges | PPI enrichment p-value |
| --- | --- | --- | --- | --- | --- | --- |
| Module 13 (darkmagenta) | 62 | 838 | 27 | 0.787 | 201 | < 1.0x 10^-16^ |
| Module 14 (darkred) | 74 | 833 | 22.5 | 0.79 | 198 | < 1.0x 10^-16^ |
| Module 19 (darkgrey) | 68 | 225 | 6.62 | 0.745 | 142 | 8.59 x 10^-11^ |
| Module 23 (white) | 66 | 302 | 9.15 | 0.783 | 133 | < 1.0x 10^-16^ |
| Module 27 (paleturquoise) | 64 | 1142 | 35.7 | 0.876 | 188 | < 1.0x 10^-16^ |
| Module 30 (cyan) | 99 | 766 | 15.5 | 0.663 | 306 | < 1.0x 10^-16^ |
