# Supplementary material for: Identification of key modules and hub genes for small-cell lung carcinoma and large-cell neuroendocrine lung carcinoma by weighted gene co-expression network analysis of clinical tissue-proteomes
Source: PLoS One. 2019 Jun 5;14(6):e0217105. doi: 10.1371/journal.pone.0217105 (PMC6550379; doi:10.1371/journal.pone.0217105)
Supplement: S4 Table — (DOC) [file pone.0217105.s005.doc]

**S4 Table.** **Top 20 genes in network ranked by Degree method.**

| **13 (darkmagenta)** | | | **14 (darkred)** | | | **19 (darkgrey)** | | | **23 (white)** | | | **27 (paleturquoise)** | | | **30 (cyan)** | | |
| --- | --- | --- | --- | --- | --- | --- | --- | --- | --- | --- | --- | --- | --- | --- | --- | --- | --- |
| Rank | Name | Score | Rank | Name | Score | Rank | Name | Score | Rank | Name | Score | Rank | Name | Score | Rank | Name | Score |
| **1** | **POLR2A** | **48** | **1** | **RPS12** | **43** | **1** | **HDAC1** | **16** | **1** | **HNRNPA1** | **20** | **1** | **RPL7A** | **53** | **1** | **RPSA** | **37** |
| **2** | **GTF2F1** | **43** | **1** | **RPL7A** | **43** | **2** | **HDAC2** | **15** | **2** | **HNRNPM** | **15** | **2** | **RPL35** | **48** | **1** | **RPS2** | **37** |
| **3** | **POLR2F** | **42** | **3** | **SNRPD2** | **39** | **2** | **SMARCA5** | **15** | **2** | **EIF3I** | **15** | **2** | **RPL36** | **48** | **1** | **RPLP0** | **37** |
| **3** | **POLR2B** | **42** | 4 | EIF4G1 | 34 | 4 | RBBP4 | 14 | 4 | EIF3E | 14 | 2 | RPS7 | 48 | 4 | RPL10A | 35 |
| 5 | POLR2C | 41 | 4 | RPS3 | 34 | 5 | SMARCA1 | 13 | 4 | EIF3D | 14 | 5 | RPL18A | 47 | 4 | RPS23 | 35 |
| 6 | POLR2L | 40 | 6 | RPL26 | 33 | 6 | SRSF2 | 12 | 4 | EIF3A | 14 | 5 | RPS2 | 47 | 6 | RPS3 | 34 |
| 6 | POLR2I | 40 | 6 | EIF4E | 33 | 7 | SRSF6 | 11 | 7 | EIF3J | 13 | 5 | RPL9 | 47 | 6 | RPS9 | 34 |
| 6 | POLR2D | 40 | 6 | RPL36 | 33 | 7 | CAD | 11 | 7 | EIF3B | 13 | 5 | RPL11 | 47 | 6 | RPL12 | 34 |
| 9 | POLR2K | 39 | 9 | RPS16 | 32 | 7 | SRSF1 | 11 | 7 | EIF3C | 13 | 5 | RPL18 | 47 | 9 | RPS14 | 33 |
| 9 | POLR2E | 39 | 9 | RPL18 | 32 | 7 | SRSF9 | 11 | 7 | EIF3G | 13 | 5 | RPL17 | 47 | 9 | RPS15A | 33 |
| 9 | POLR2H | 39 | 9 | RPL12 | 32 | 11 | SRSF7 | 10 | 7 | EIF3M | 13 | 5 | RPS16 | 47 | 9 | RPS6 | 33 |
| 12 | HNRNPA1 | 38 | 9 | RPLP0 | 32 | 11 | BAZ1A | 10 | 7 | EIF3L | 13 | 5 | RPL10A | 47 | 12 | RPS3A | 32 |
| 12 | POLR2G | 38 | 9 | RPS14 | 32 | 11 | HNRNPUL1 | 10 | 7 | EIF3F | 13 | 5 | RPS13 | 47 | 12 | RPS15 | 32 |
| 12 | POLR2J | 38 | 9 | RPL10A | 32 | 11 | SRSF3 | 10 | 7 | EIF3K | 13 | 5 | RPL29 | 47 | 12 | RPS7 | 32 |
| 15 | HNRNPU | 37 | 9 | RPL31 | 32 | 11 | BPTF | 10 | 7 | EIF3H | 13 | 5 | RPS15 | 47 | 12 | RPS11 | 32 |
| 16 | PTBP1 | 36 | 9 | RPS5 | 32 | 11 | U2AF2 | 10 | 7 | PRPF19 | 13 | 5 | RPS3 | 47 | 12 | RPS18 | 32 |
| 16 | RBMX | 36 | 9 | RPS6 | 32 | 11 | SF3A2 | 10 | 7 | EIF3CL | 13 | 5 | RPL23A | 47 | 12 | RPS13 | 32 |
| **16** | **GTF2F2** | **36** | 18 | RPL13A | 31 | 18 | UBA52 | 9 | **7** | **U2AF2** | **13** | 5 | RPL8 | 47 | 12 | RPS5 | 32 |
| 16 | HNRNPD | 36 | 18 | RPL13 | 31 | 18 | BAZ2A | 9 | 19 | PCNA | 12 | 5 | RPL30 | 47 | 12 | RPL18A | 32 |
| 20 | HNRNPH1 | 35 | 18 | RPL8 | 31 | 18 | RUVBL1 | 9 | 19 | HDAC2 | 12 | 5 | RPS5 | 47 | 12 | RPS16 | 32 |
